# Maturing global CO_2_ storage resources on offshore continental margins to achieve 2DS emissions reductions

**DOI:** 10.1038/s41598-019-54363-z

**Published:** 2019-11-29

**Authors:** P. S. Ringrose, T. A. Meckel

**Affiliations:** 1 Department of Geoscience and Petroleum, Norwegian University of Science and Technology, Trondheim, Norway; 2 Equinor Research and Technology, Trondheim, Norway; 3 Gulf Coast Carbon Center, Bureau of Economic Geology, The University of Texas at Austin, Austin, Texas USA

**Keywords:** Climate-change mitigation, Hydrology

## Abstract

Most studies on CO_2_ emissions reduction strategies that address the ‘two-degree scenario’ (2DS) recognize a significant role for CCS. For CCS to be effective, it must be deployed globally on both existing and emerging energy systems. For nations with large-scale emissions, offshore geologic CO_2_ storage provides an attractive and efficient long-term strategy. While some nations are already developing CCS projects using offshore CO_2_ storage resources, most geographic regions have yet to begin. This paper demonstrates the geologic significance of global continental margins for providing broadly-equitable, geographically-relevant, and high-quality CO_2_ storage resources. We then use principles of pore-space utilization and subsurface pressure constraints together with analogs of historic industry well deployment rates to demonstrate how the required storage capacity can be developed as a function of time and technical maturity to enable the global deployment of offshore storage for facilitating 2DS. Our analysis indicates that 10–14 thousand CO_2_ injection wells will be needed globally by 2050 to achieve this goal.

## Introduction

## The Role of CCS in the Energy Transition

A major societal challenge is achieving globally significant reductions in greenhouse gas emissions to the atmosphere. There is growing clarity from numerous studies^1–3^ that large-scale geologic disposal of CO_2_ from industrial emissions will be essential to achieve this objective. The ‘wedge model’ analysis for identifying opportunities for CO_2_ atmospheric reductions^4^ remains useful for anticipating contributions from different sectors – essentially a blend of growth in renewable energy use, improved energy efficiency, and various means of decarbonization of energy production and consumption. In this construct, CO_2_ Capture and Storage (CCS) is anticipated to support approximately 13% of total cumulative emissions reductions through 2050, requiring around 120,000 million tones (Mt) of cumulative CO_2_ reduction by 2050. Annual storage rates in 2050 are expected to be 6–7,000 Mtpa^5^. Furthermore, the IPCC argue that emissions reduction costs without CCS deployment could be as much as 29% to 297% higher by 2100^6^. Lastly, many sectors of the modern economy, such as cement and steel production, are dependent on CCS alone to achieve significant decarbonization.

Despite this widespread recognition of the important role of CCS, fundamental doubts seem to remain among communities and policy makers about the viability and effectiveness of CCS deployment. There is certainly a significant economic hurdle, but active projects do exist and costs are decreasing with technology maturation, such that full-chain (capture, transport, storage) CCS can currently be considered as technically demonstrated and available as an integrated decarbonization technology (Norway, Japan, and Brazil have active offshore CO_2_ injection projects and the UK, USA, Australia and China have projects in the planning stages).

A recent assessment of the long-term performance and security of CO_2_ storage indicates a high degree of confidence in retention^7^. Despite some skepticism about project deployment, there are currently 19 CO_2_ injection projects globally^8^, of which 4 large-scale projects are dedicated to geologic storage in saline formations (Sleipner, Snøhvit, Quest, IBDP) which together inject nearly 4 million tonnes CO_2_ per annum (Mtpa). The 19 large-scale CCS facilities in operation together with a further 4 under construction, have an installed capture capacity of 36 Mtpa^8^. Additional experience in handling, transport, and injection of CO_2_ has been gained from almost fifty years of enhanced oil recovery (CO_2_ EOR). CCS is therefore demonstrated and underway at industrial scales globally; however, an order of magnitude increase is needed to meet the long-term expectations for CCS and to realize the 2DS goals.

In this paper we reinforce the overall viability of CCS and propose a meaningful timeline by using the historic perspective of the utilization of hydrocarbon resources in sedimentary basins as an analog to demonstrate the future utilization of the same basin geologic resources for CO_2_ disposal. Our conclusions offer decision makers a rational perspective for further support to allow CCS to deliver on stated emissions reduction goals. As our focus is on deep subsurface geological storage of captured CO_2_ (GCS), we will refer to GCS as the principal objective, assuming that significant global CO_2_ capture activities emerge in parallel.

## Gigatonne-Scale CO_2_ Storage in Offshore Basins

Our analysis is based on the broad similarities in the stratigraphic and tectonic histories of passive continental margins and clarifies the primary factors affecting basin-wide and global storage potential (capacity), emphasizing typical subsurface fluid pressure profiles. Important local and regional differences in the tectonic histories of the continental margins are discussed in the Appendix (See Appendix: Methods used in supporting the main paper). Our approach departs from extensive prior regional volumetric quantification techniques^9^ that rely on a subsurface volumetric efficiency factor (ε). Rather, we develop concepts that emphasize regional stratigraphic pressure constraints that will matter at the Gigatonne (Gt) storage scale, referred to here as the ‘basin ΔP’ approach. We then demonstrate, using historic industry hydrocarbon well development data at three different regional scales, combined with rational average injection rates informed by the stratigraphic pressure analysis and practical experience, that accessing this storage resource is possible on the required timeframes and within pressure constraints that allow GCS to deliver the expected emissions mitigation role. We also argue that the history of technology development in extracting oil and gas resources over the last century (termed the primary, secondary and tertiary recovery methods) can to some extent be applied for evaluating similar future phases of CO_2_ disposal technology, each employing more advanced pressure management methods. Our aim is to provide the first-order technical basis and confidence that various nations need to effectively and simultaneously develop their offshore geology for GCS on a timeline that is relevant for 2DS^10^.

**Figure 1:**
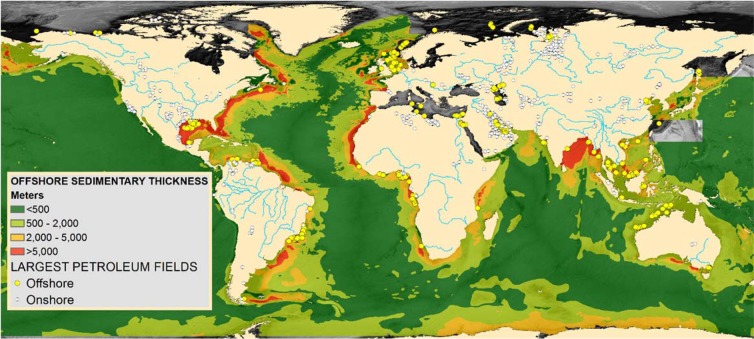
Map of global distribution and thickness of sediment accumulations on continental margins^52,53^ with the thickest stratigraphy indicated in red. Yellow dots represent the largest offshore hydrocarbon fields (i.e. suitable large-scale subsurface hydrocarbon containment demonstrated^54^, and blue lines are the largest continental river systems, often leading to extensive and thick offshore Cenozoic stratigraphy.

**Figure 2:**
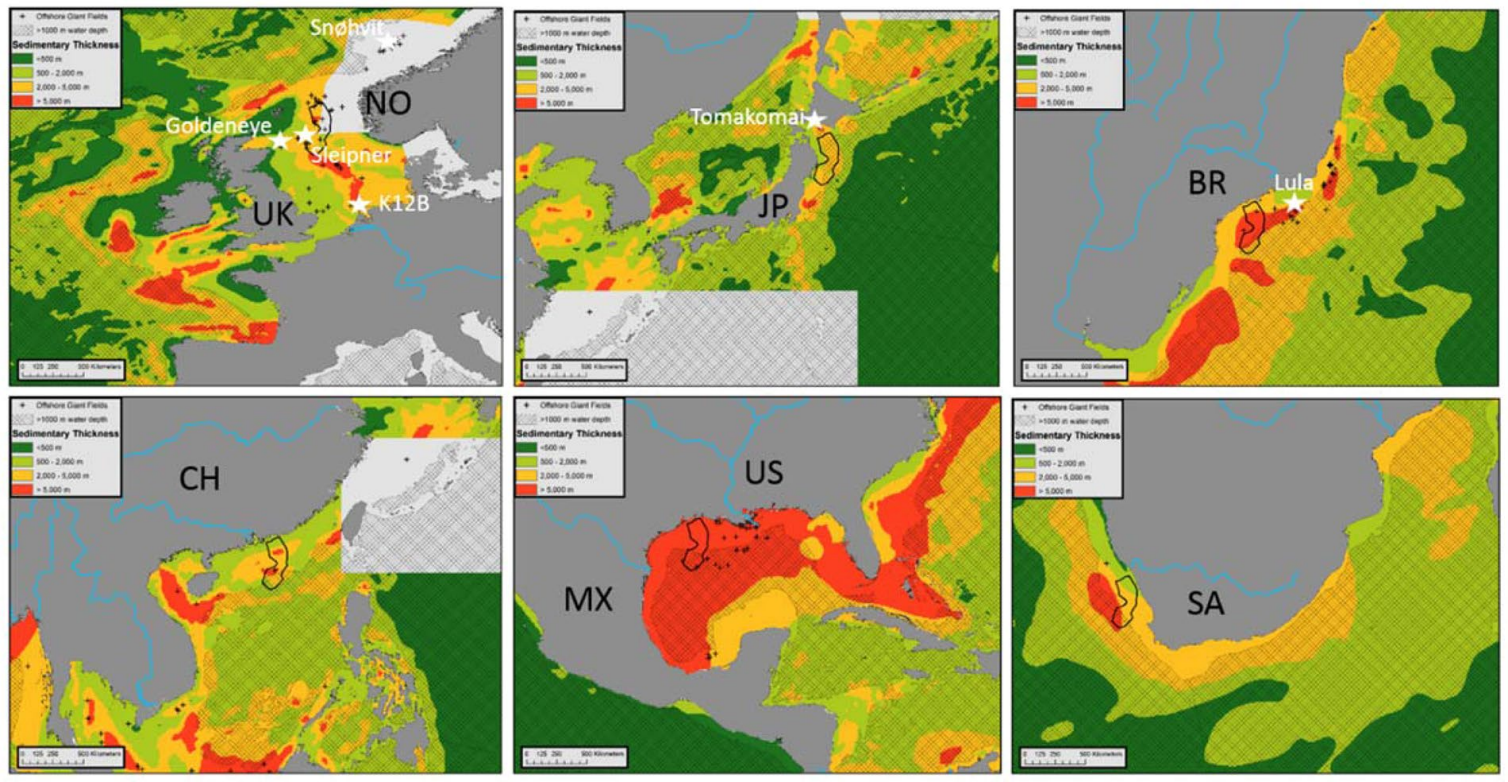
Comparison of prospective storage resource regions for selected global localities at the same map scale (1:15,000,000). The footprint of the Utsira sandstone formation (North Sea) utilized for GCS since 1996 is indicated in the solid black outline and represents the size of a typical regional geologic storage target. Currently active (Sleipner, Snøhvit, Tomakomai, Lula), completed (K12B) and proposed (Goldeneye) offshore CO_2_ injection projects are indicated with white stars. Cross-hatched regions have water depths >1,000 m. Major hydrocarbon fields (Fig. 1) are shown in black cross symbols, indicating favorable conditions for large-scale subsurface retention of buoyant fluids.

The global offshore continental shelves (Fig. 1) represent the most significant Gt-scale storage resource for GCS. Onshore basins are also important, but the offshore settings offer both significant volumes and practical deployment benefits at scale. Offshore continental margins, dominated by thick Cenozoic-age sediments provide vast subsurface rock volumes broadly prospective for storage due to their suitable subsurface depth range and relatively young age (low compaction, limited diagenesis, and high porosity). This volumetrically-significant resource can adequately and efficiently match the global objective of Gt-scale CO_2_ disposal. Furthermore, this resource benefits from lower technical and societal risks related to regionally-limited access to suitable onshore geology (e.g. EU, Atlantic US, China and India), issues related to protection of potable groundwater resources, and avoidance of population centers. The existence and historic exploitation of numerous giant hydrocarbon accumulations in offshore basin settings (Fig. 1, yellow symbols) can also be taken as evidence for appropriate subsurface conditions for retention of large volumes of buoyant non-wetting fluids over geologic time scales, giving an excellent precedent for successful deployment of GCS. Regional comparison of the overall similarity of geographic extent of available storage resources for select regions is provided in Fig. 2, illustrating a broadly equitable storage and geographically relevant resource potential.

Many offshore continental margin basins (Figs. 1 and 2) have comparable geologic evolution that has resulted in broadly similar stratigraphic and structural elements: typically, a phase of continental rifting and subsidence followed by a period of passive margin coastal progradation^11,12^. Decades of investigation indicate that these margins exhibit a deeper rift sequence with some variability in structural style^13^, typically Mesozoic in age (often representing the rifting of the Pangaea supercontinent around 175 million years ago), with advanced diagenesis (cementation and porosity reduction due to burial and interaction with hot fluids). These deeper rift sequences are typically overlain by net-progradational and aggradational Cenozoic sediments composed of fluvial, deltaic, shelf, and slope deposits. Where large continental-draining river systems enter these settings (Fig. 1, blue lines), clastic accumulations may exceed many kilometers thickness. Other margins may have extensive carbonate development^14^, also suitable for GCS. These Cenozoic-age passive margin deposits are also characterized by lower levels of diagenesis (porosity reduction) and less pervasive faulting than the underlying Mesozoic stratigraphy. Arguably, the deeper rift sequences are less well-suited for the first phase of GCS, while the upper Cenozoic sequences offer some of the best regional saline aquifer storage targets (such as the Utsira sandstone offshore Norway; Fig. 2). In all cases, the essential storage requirement is to find thick high-porosity sediment reservoir units overlain by sealing units (usually thick shales), ideally with open hydrologic systems for dissipating induced pressure increases. Shallower projects also have reduced drilling costs. The deeper rift sequence often includes many large-scale faults that often propagate upwards and generate additional subsequent faulting in the overlying stratigraphy. The fault architecture is also a critical element for storage site characterization, since faults can both transmit and retain buoyant fluids^15,16^. In a global petroleum assessment, 71% of the known hydrocarbon reserves occurred in structural (i.e. faulted) traps, as opposed to stratigraphic or other trap types^17^, suggesting faults are commonly involved in high-saturation subsurface buoyant fluid retention.

## Basin-Fluid Pressure Analysis Approach

Two of the three largest global hydrocarbon (oil and gas) producing regions are the North Sea and the Gulf of Mexico – the largest being the Middle East/Persian Gulf region, which is mainly onshore and partly offshore. We will therefore consider these two basins as representative of a mature state of subsurface knowledge for the highly prospective offshore basins available for large-scale CO_2_ disposal. One of the most significant common features in geologic development of the continental margins is the subsurface fluid pressure distribution^18^. Typically, these geologic basins have a shallow interval (<2–3 km) with hydrostatic (normal) pressures that develop with depth into naturally over-pressured systems, a common feature which can be deduced from the initial reservoir pressure data from decades of hydrocarbon exploration in different basins^19–23^. This behaviour is essentially controlled by a natural balance between the rate of compaction and the rate of fluid pressure dissipation^24^, where a loss of balance is usually termed ‘disequilibrium compaction’, although other processes are also involved in generating overpressure^25^.

**Figure 3:**
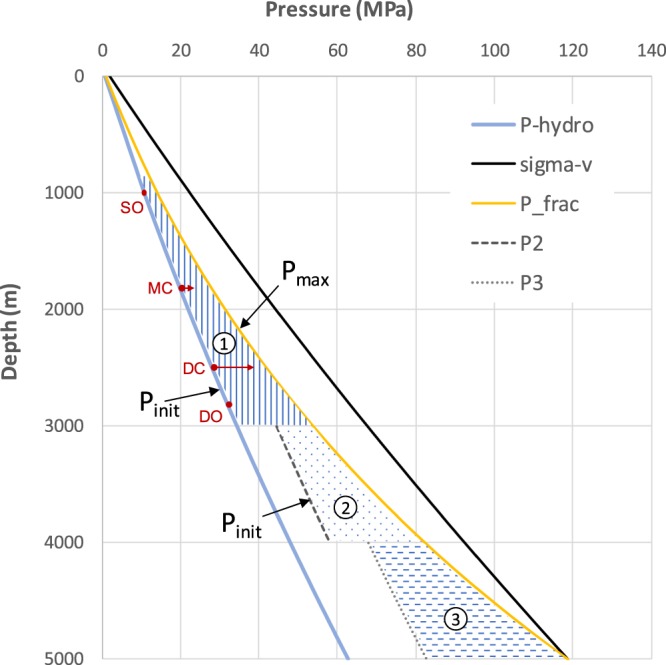
Pressure depth functions for a generalised Norwegian North Sea basin case illustrating the shallow normally pressured region (1), and the progressively deeper and more overpressured regions (with excess initial pressure P2 and P3). P-hydro is the hydrostatic gradient, sigma-V is the vertical principal stress, and the maximum reservoir pressure is described by the formation fracture pressure P_frac (See Appendix: Methods used in supporting the main paper).

As the fluid pressure increases with depth it begins to approach the lithostatic pressure gradient, and a limiting pressure is reached – the rock fracture pressure – such that subsurface reservoirs rarely exhibit pressures greater than 80–90% of the lithostatic pressure (often taken to be 22.6 kPa/m or 1 psi/ft). This general behavior is characterized in the depth plot shown in Fig. 3, based on a generalized Norwegian North Sea basin case. For comparison, average initial-reservoir pressure trends for all Miocene reservoirs on the inner shelf of Texas^26^ are shown to be hydrostatic to approximately 2,750 meters^27^, consistent with regional GoM data^28^. Three depth zones are identified in this generic plot: (1) a normally pressured zone between 1 and 3 km depth, (2) a weakly over-pressured zone between 3 and 4 km depth, and (3) a high over-pressured zone between 4 and 5 km depth. The actual depths of these zones and style of vertical transition will be basin dependent, but the trend with depth is commonly observed. Referring to the stratigraphic summary above, the Cenozoic sequences are typically in the normally-pressured zone, while the deeper Mesozoic rift-sequences are commonly found in the deeper over-pressured zones (with many exceptions to that simplification).

Appreciation of this common fluid pressure trend with depth (Fig. 3) is arguably the single most significant consideration for offshore global GCS deployment at the Gt-scale in a reasonable timeframe (assuming that the basic reservoir and seal characteristics are identified^29^). This is because large-scale CO_2_ disposal will require subsurface pressure management, rather than being simply controlled by the available subsurface pore volumes. While pore volume is a static metric, pressure evolution involves time, which is an important consideration for understanding how GCS can meet intended volumetric targets within anticipated timelines through mid-century. This has been described as ‘dynamic capacity’^30^, and while pressure constraints have been identified and discussed previously as key factors for CCS^31–34^, this evaluation has primarily been considered for reservoir-scale performance^35^ rather than at a stratigraphic scale.

Reservoir pressure mitigation methods involving subsurface brine extraction have also been investigated^36^, but ultimately include re-injection into another nearby stratigraphic interval, which does not overcome the large-scale stratigraphic pressure limitations that are considered here. So, while brine extraction and re-injection may enable single projects to be optimized, the strategy is not necessarily favorable for long-term Gt-scale storage in a basin employing multiple projects throughout the stratigraphy and may not be required. However, pressure management among multiple projects may be useful in the later stages of storage resource development (as argued below).

Thus, while CO_2_ storage trapping mechanisms^37^ (structural trapping, residual-phase trapping, dissolution and mineralization) are essential to GCS, it is the subsurface reservoir pressure that ultimately limits CO_2_ injection and the total storage capacity at the Gt-scale at operational timescales. Pressure propagates in the subsurface far more effectively and pervasively than injected fluids, and the pressure footprint can be assumed to extend outward from an injection well by a factor of 10 to 100 compared to the dimensions of the CO_2_ plume^38^. The importance of pressure limitations was encountered at the Snøhvit project in the Barents Sea (a Mesozoic injection target), where the initial injection well had to be modified to allow access to stratigraphic units with better pressure communication^39,40^. As a corollary, the Sleipner project (Cenozoic) has not encountered any pressure limitations, being connected to a large open aquifer system. Here, we develop the concept of the ‘available pressure resource’ for global deployment of offshore GCS, using the cases of the North Sea and the Gulf of Mexico as a reference.

Our proposed generic approach, the ‘basin ΔP’ approach, is based on integration of the injectivity equation over the project lifetime, where pressure limits are defined by basin pressure. We obtain the following function (See Appendix: Methods used in supporting the main paper):

1 $${V}_{project}={I}_{C}[{p}_{well}-{p}_{init}+{\int }_{i}^{f}A{p}_{D}({t}_{D})]+{F}_{b}$$

where,

V_project_ is the estimated volume stored

I_c_ is the injectivity

P_well_ is the injection well pressure

P_init_ is the initial reservoir pressure

Ap_D_(t_D_) is a characteristic pressure function

F_b_ is a volume flux boundary condition.

**Figure 4:**
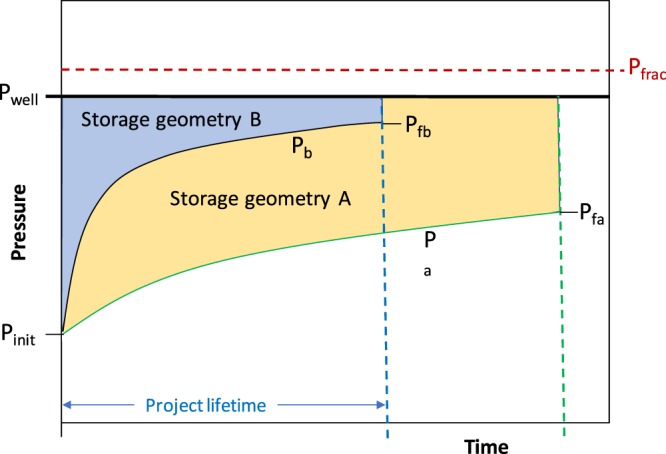
Idealised project lifetime pressure plots for two contrasting aquifer units assuming the same initial pressure conditions.

The characteristic pressure function, the integral of reservoir pressure with time, is a function of the formation properties and the dimensions of the storage unit, represented graphically in Fig. 4. The integration is between the limits P_init_ and P_final_, where P_final_ may be defined with reference to p_frac_ as a limiting condition. For a closed saline aquifer unit with no-flow boundary conditions (such as a sealed fault block), F_b_ = 0; while for the case of some pressure dissipation from the saline aquifer formation, F_b_ is positive, and for a case with some brine influx into the storage unit F_b_ is negative. It is generally assumed that F_b_ is a small factor compared to the injectivity term. However, for the case of an infinite aquifer with no pressure boundary limitation, F_b_ could be large or even dominant.

Equation (1) assumes a constant injection pressure and constant injectivity – simplifying assumptions appropriate for screening prospective projects and evaluating expected GCS performance. With more complex operational variables, numerical reservoir simulation can be used to more accurately assess injection volumes as a function of variable pressure gradients. At the project screening stage, parameters for Eq. (1) can be estimated using regional basin data (See Appendix: Methods used in supporting the main paper) and initial estimates of storage unit geometry and formation permeability. Volumes may be converted to mass using estimates for the mean *in situ* CO_2_ density. An illustration of the application of Eq. (1) to a real dataset is given in the Appendix (See Appendix: Methods used in supporting the main paper).

There are essentially two operational criteria for stopping storage projects:

A. The storage project fills the available pore-space before the maximum pressure limit is reached (Aquifer geometry A in Fig. 4);
B. The storage project reaches the maximum pressure limit before the available pore-space can be fully utilized (Aquifer geometry B in Fig. 4).

The concept outlined in Fig. 4 is scaled to a common set of initial conditions: the initial reservoir pressure, P_init_, the bottom-hole well pressure, P_well_, and the formation fracture pressure P_frac_. Storage geometry A follows a pressure path P_a_ towards a final pressure P_fa_, and likewise for B.

The Sleipner and Quest projects are examples of A, while the early injection history at the Snøhvit project was an example of B. A further situation is also possible where CO_2_ is injected in an inclined aquifer, with lateral migration gradually being hindered and eventually stopped by processes of structural, residual and solubility trapping^41,42^. This would be a variant of A, since pressure would not be a limiting factor.

Thus, in general, if all prospective storage formations fall into either of these two categories, the total storage resources will be smaller than the initial static volumetric estimates based on storage efficiency, ε, since the B-category aquifers will be pressure limited. As we argue below, early projects will tend to focus on the best available storage opportunities provided by ‘Class A’ aquifers (and in the offshore basins these will typically be found in the shallower mainly post-rift Cenozoic stratigraphy). As the global need to access storage resources grows, projects will then start to exploit the ‘Class B’ aquifers, having to adjust project designs to cope with the local formation pressure limits. A third class of storage projects, which we will term ‘Class C’ will be the cases where active pressure management is used to further enhance storage availability. This will allow natural pressure limits to be circumvented by active production schemes, including brine production^43^ the use of the ‘pressure space’ created by oil and gas production^44^ or direct injection into depleted gas fields^45^. We argue that this transition from early use of CO_2_ injection into aquifers without significant pressure limits (Class A), through to CO_2_ storage in pressure-limited aquifers (Class B) and eventually to pressure management at the basin scale (Class C), represents a global technology development strategy for storage (Table 1), which is analogous to the historic oil and gas production strategy which has moved from primary recovery (pressure depletion modus), to secondary recovery technology (mainly pressure management by water injection), and then to tertiary methods (involving injection of gas, CO_2_ and other chemicals to further enhance hydrocarbon recovery). We know from historic data that each new phase of oilfield recovery added a factor of 0.5–1 to the previously recoverable oil resources.

**Table 1:** Comparison of historic oil and gas recovery strategies with the proposed CO_2_ storage resource.

Oil and gas domain	Primary production	Secondary recovery	Tertiary recovery
Recovery mechanisms used	Pressure depletion	Pressure support (mainly waterflood)	Gas & CO_2_ injection, chemical flooding
**Typical recovery factor**			
(% HCIP)	<30%	30 to 50%	40 to 80%
**CO**_**2**_ **storage domain**	**Class-A projects**	**Class-B projects**	**Class-C projects**
Pressure management approach	Projects with minimal pressure constraints	Projects constrained by pressure limits	Projects with active pressure management
Typical pore space utilized (% Pore Volume)	<6% of open aquifer systems	<4% of confined aquifer systems	>5% for targeted confined aquifer systems

**Figure 5:**
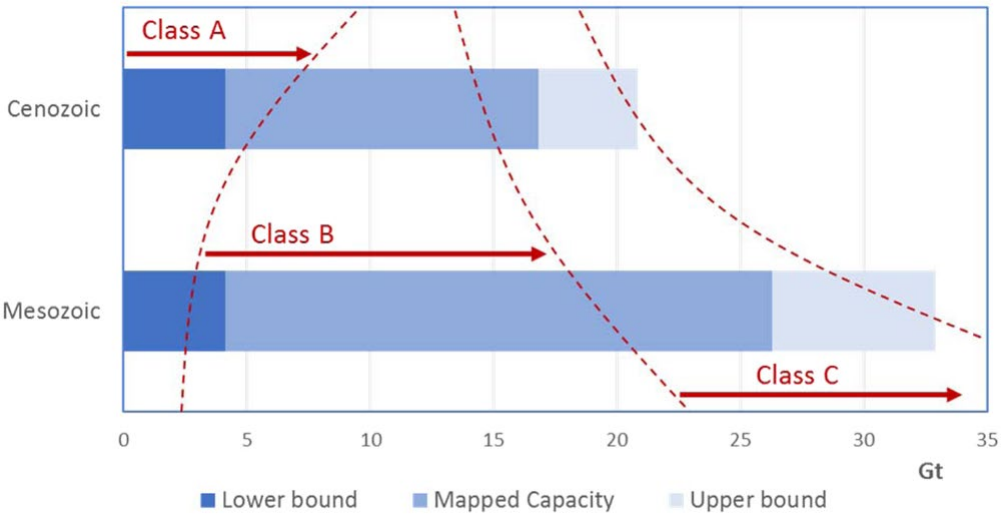
CO_2_ storage resource development strategy, illustrated for the case of the mapped Norwegian North Sea resource base. Here we have used the lower-bound resource estimate to identify the Class A resource with a preference for initial deployment in the shallower Cenozoic stratigraphy. The Class B resource  approaches the mapped capacity values and utilizes deeper stratigraphy. Class C is used to exploit the upper bound potential in the last phase.

It is not simple to predict how successive stages of technology development will work to increase the accessible CO_2_ storage resources, but as an indicator of this potential we can use the relatively mature storage resource assessments for the Utsira formation offshore Norway^46^. For the Utsira Fm. structural trapping of free-phase CO_2_ (a Class-A resource) provides ~0.8 Gt of storage^47^, while injection up to the natural pressure limits (Class-B resource) could allow up to 8.3 Gt of storage^48^. Studies of the potential Utsira storage resource when deploying active pressure management (Class-C resource) gave estimates between 42 and 50 Gt of storage^47,49^. The potential for growth in storage resources as a function of increasing application of technology is therefore significant. This strategy for utilization of the global offshore basin storage resource is captured graphically in Fig. 5.

To illustrate the range of likely behaviour of different CO_2_ injection projects at different stratigraphic depths and contrasting reservoir conditions, we postulate four model scenarios (also shown on Fig. 3):

- A shallow open-boundary case (SO) with injection at 1000 m depth and with no significant pressure constraint (a Cenozoic Class-A resource);
- A moderate-depth, partially-closed pressure boundary case (MC) with injection at 1800m depth (a Cenozoic Class-B resource);
- A deep closed-boundary case (DC) with injection at 2500 m depth (a Mesozoic Class-B resource);
- A deep open-boundary case (DO) with injection at 2800 m depth and with no significant pressure constraint (a Mesozoic Class-A resource).

These have been modeled using Eq. (1), with the assumptions shown in Table 2,  to estimate the storage metrics and have well rates that cover the observed range in historical injection data (See Appendix: Methods used in supporting the main paper). Of course, a wide range of scenarios are possible – these are only intended to portray representative well behaviors. Of these 4 scenarios, the DC case reaches a pressure limit before the end of the expected well life of 25 years, resulting in only 5.1 Mt stored at project closure in year 16. The best case (SO) achieves 23.4 Mt stored after 25 years, and the mean injection rate for all four cases is 0.57 Mtpa, close to the historical mean of 0.53 Mtpa and lower than the historical mean for offshore wells at 0.7 Mtpa (See Appendix: Methods used in supporting the main paper).

## Global CO_2_ Injection Development well Rate and Timeline

Given the reservoir performance concepts developed above, and the constraint of expected average injection rates, how then could a strategy for systematic use of this subsurface offshore continental margin stratigraphic storage resource be implemented? We address this by considering the history of hydrocarbon industry development in the selected regions to provide a template for a credible deployment timescale for CCS as an analog for achieving global emissions reduction targets.

**Figure 6:**
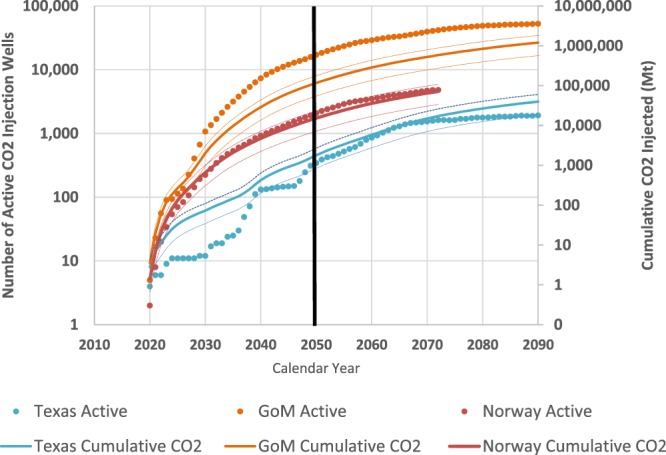
Projected growth of the number of CO_2_ injection wells and the cumulative CO_2_ injected, based on historical hydrocarbon well development for three different geologic regions. Historic datasets have been replotted beginning in 2020 to provide a perspective on potential future regional CCS well deployment. The lower slope of the data in late years is a result of hydrocarbon production maturation (resource depletion, creaming concepts), which might also occur with CO_2_ storage when resource limits are eventually reached (volume or pressure). Thin dashed lines represent high (P10) and low (P90) bounds (See Appendix: Methods used in supporting the main paper) based on injection rates of 0.33 and 1.06 Mta.

Figure 6 presents future well-development based on historical well performance for the Texas inner shelf of the Gulf of Mexico, the entire Gulf of Mexico, and the Norwegian North Sea (well count data from Texas Railroad Commission, U.S. Bureau of Safety and Environmental Enforcement, and the Norwegian Petroleum Directorate). The primary data provided are the annual and total cumulative number of wells drilled in each region. The historical curves have each been shifted such that the initial year for the first well is 2020 (although a few offshore CO_2_ injection wells existed globally before that time). The cumulative number of historic hydrocarbon wells has been translated to storage volumes assuming a 25-year well life for each CO_2_ injection well, a reasonable assumption given the experience with enhanced oil recovery using CO_2_ injection wells in the Permian Basin of west Texas. This results in some fall-off of active well numbers in the years after 2050. The number of wells active in 2050 in these scenarios are 17,155 for the Gulf of Mexico case, 2,083 for Norway and 345 for Texas.

The mean CO_2_ injection rate per well is assumed to be 0.7 Mtpa (or 17.5Mt per well over the 25-year lifetime), based on the available data for injection rates to date (See Appendix: Methods used in supporting the main paper) and consistent with pressure-sensitive results derived in Table 2. Using available historic data from industrial-scale storage projects in operation (See Appendix: Methods used in supporting the main paper) we obtain rate estimates of 0.532 ± 0.271 Mtpa for all wells and 0.695 ± 0.222 Mtpa for the offshore wells only. Using the offshore wells statistics (See Appendix: Methods used in supporting the main paper) we then obtained values for a 90% confidence interval: P90 = 0.33, P50 = 0.70, P10 = 1.06 (where P90 indicates 90% probability of exceedance). We recall that the IEA^5^ envision global CCS deployment capable of capturing and storing up to 7 Gt of CO_2_ emissions per year in 2050, with total cumulative mitigation of 120 Gt at that time^50^. Using the assumed mean injection rate of 0.7 Mtpa per well, this implies that over 10,000 CO_2_ injection wells (delivering 7,000 Mt per year total) may need to be in operation by 2050. Is this plausible? Essentially yes, considering historic well development rates. For example, by converting the historical well development trajectories into future CO_2_ injection wells (Fig. 6) and assuming 0.7 Mtpa average injection rates, we can infer that:

- A single ‘Gulf-of-Mexico well development’ CO_2_ injection model could achieve the 7 Gtpa storage by 2043 and 12 Gtpa by 2050. Cumulative storage in 2050 would be 116 Gt.
- Alternatively, five ‘Norway offshore well development’ models could achieve the 7 Gtpa storage by 2050. Cumulative storage in 2050 would be 73 Gt.
- Cumulative storage of >100 Gt by 2050 is most efficiently achieved with 5–7 regions pursuing a Norwegian-scale offshore well development model using individual well injection rates between 0.5–1 Mta, although it could be achieved with a single GoM model with 0.7 Mtpa injection rates.

**Table 2:** Parameter assumptions for four injection-well model scenarios and resulting storage metrics.

Parameter	SO	MC	DC	DO
Injection Depth (m)	1000	1800	2500	2800
Formation Temperature (C)	35	63	88	98
P_initial (bar)	108.0	200.0	290.0	319.0
P_final (bar)	110.0	230.2	390.7	323.0
P_well (bar)	138.0	250.0	380.0	380.0
Injectivity (m3/day/bar)	120	80	40	30
Pressure constraint factor, A	1	15	50	2
Mean annual injection rate (Mt)	0.9	0.7	0.3	0.4
Total injected (Mt)	23.4	9.2	5.1	11.4
Project termination year	25	25	16	25

Volume to mass conversion uses standard properties for CO_2_ assuming thermal equilibrium. The average annual injection rate across the four cases is 0.57 Mtpa.

The point of this extrapolation is to demonstrate that it will only take a fraction of the historic worldwide offshore petroleum well development rate to achieve the global requirements for GCS. While offshore CCS is suitable many places (recall Figs. 1 and 2), it does not have to be deployed everywhere to achieve global benefit, and focus can be on the most prospective and economic regions. In practice, these developments would likely occur in multiple offshore basins close to the main locations of onshore capture; however, our selected basin development curves constrain the total well rate required to achieve the incremental and cumulative 2DS emissions reduction goal for 2050. Further discussion of the assumptions made in this evaluation and alternative injection wells scenarios are given in the Appendix (See Appendix: Methods used in supporting the main paper).

To obtain a preliminary cost estimate for this potential global offshore drilling programme, we note that offshore injection well costs are of order ~50–100 M€ (55–110 MUSD) per well, assuming a 2015 reference case^51^. The offshore drilling costs in terms of emissions avoided are therefore of order 2.9–5.5 €/tonne (3.2–6.3US$/tonne) for our mean well rate of 17.5Mt per well. This does not include the costs of capture, transport or platform infrastructure, but indicates that offshore saline aquifer storage can be a cost-effective emissions-mitigation measure in a world where the cost/penalty of emitting to atmosphere rises above the current level of 20–60 US$/tCO2e (carbonpricingdashboard. worldbank.org).

## Conclusions

CCS is essential for realizing a global emissions reduction strategy consistent with 2DS aspirations. Globally, it is the continental margin geology that can most rapidly accommodate the large-scale CCS anticipated. There are many well-established global geologic similarities in these basins, and prior petroleum exploration provides an exceptionally well-documented starting point for deploying CCS in these settings. We propose using the characteristic pressure and stress versus depth trends in these basins as a framework for determining the initial and final pressure bounds (the basin ΔP approach) for determining the capacity of prospective storage projects. By utilizing the ‘pressure stratigraphy’ of these basins, early class-A projects can exploit the most accessible storage sites (generally shallower and less constrained by pressure limits), while later projects will exploit the majority of sites (class-B projects) which will have practical pressure limits governed by the basin stress and pressure profiles. Eventually, more advanced technology using pressure management approaches (class-C projects) will allow further resource development. This forward strategy for CCS has a precedent in the historic development of technology in oil and gas projects.

The timeframe of Gigatonne-scale CCS is hard to evaluate using either multiple individual numerical reservoir simulations (too many are needed) or using regional static volumetric assessments (which are likely optimistic as they don’t account for the dynamic pressure conditions). However, by developing a basin-scale pressure model to frame project capacity assessments, we propose a consistent and transparent basis for assessing and developing these resources.

Using historic well development scenarios from mature hydrocarbon basins and applying stratigraphic dynamic pressure constraints, a strategy for accessing the required storage capacity through time is demonstrated, providing a roadmap for global deployment of offshore CO_2_ storage consistent with the 2DS objective. Using this analysis, it is clear that the required well rate for realizing global CCS in the 2020–2050 timeframe is a manageable fraction of the historical well rate deployed from historic petroleum exploitation activities and is most efficiently achieved with multiple simultaneous regional developments.

## Supplementary information

Supplementary_Information_Appendix

## Data Availability

Methods used are described in the Supplementary Information (Appendix).
