# Supplementary material for: Maturing global CO2 storage resources on offshore continental margins to achieve 2DS emissions reductions
Source: Sci Rep. 2019 Nov 29;9:17944. doi: 10.1038/s41598-019-54363-z (PMC6884532; doi:10.1038/s41598-019-54363-z)
Supplement: Supplementary file 1 — Supplementary_Information_Appendix [file 41598_2019_54363_MOESM1_ESM.docx]

# Appendix: Methods used in supporting the paper “Maturing global CO_2_ storage resources on offshore continental margins to achieve 2DS emissions reductions”

P. S. Ringrose^1,2*^ and T. A. Meckel^3^

^1^ Department of Geoscience and Petroleum, Norwegian University of Science and Technology, Trondheim, Norway

^2^ Equinor Research and Technology, Trondheim, Norway

^3^ Gulf Coast Carbon Center, Bureau of Economic Geology, The University of Texas at Austin, Austin, Texas, USA

*Corresponding author email phiri@equinor.com

## A1 Offshore Basins – methods and uncertainties

It is very challenging to summarize the cumulative efforts over several decades in characterization and understanding the geologic development of offshore sedimentary basins. Broad patterns are predictable, while specific elements may differ depending on basin history. However, to reinforce our generalizations we briefly synthesize prior work and illustrate the character of six selected basins based on published studies (Figure A1). One of the main achievements of stratigraphic studies since the 1970’s has been the development of sequence stratigraphic concepts that allowed interpretation of passive continental margin stratigraphy in the context of global relative sea level variation^1^, and the subsequent application of those predictive concepts in subsurface seismic stratigraphic interpretation^2,3^ allowing broad stratigraphic comparisons to be made among different basins. The effectiveness of this understanding was exemplified by rapid methodical expansion of global hydrocarbon exploration. Such exploration efforts have provided an advanced understanding of subsurface fluid pressure distributions, and the geologic conditions favorable for retaining hydrocarbon accumulations^4,5,6^. Observed similarities between basins led to concepts for classifying the basin hydrodynamic and petroleum systems, providing important concepts that also apply to GCS, such as rates of vertical and lateral drainage, the development and dissipation of overpressure^7^ and the nature of petroleum migration and retention^8,9^. Over a similar timeframe, the global subsurface stress distribution has been cataloged globally using extensive well data^10^. These are not new findings, which is exactly the point: GCS can evolve from a very mature understanding of geologic, tectonic, and fluid history. This advanced level of integration of stratigraphy, fluid pressure, and stress fields provides an exceptional technical basis for pursuing gigatonne-scale CCS. These continental margin settings have retained tremendous volumes of hydrocarbon resources (Fig. 1; Ref ^11^), providing a strong indication of highly suitable subsurface geologic conditions for Gt-scale CO_2_ disposal.

There are certainly important basinal differences: some basins have significant salt tectonic components (Brazil, Gulf of Mexico), others are seismically active (Pacific USA, southeast Asia), and yet others at high latitudes have significant geologically-recent vertical tectonic components due to Quaternary glacial cycles (e.g. the North Sea^12,13^). The timing of the main rifting phase may also vary significantly, such as offshore NW Australia^14^. Additionally, not all continental margins are passive, with those on the Pacific Rim being more tectonically active (compressional, translational). Furthermore, not all Cenozoic extensional settings have experienced the same extensional rates, durations, and magnitudes, which contribute to significant basinal differences, mostly in structural style but also in stratigraphic thickness (isopach). The provenance (source geology) of clastic sediments can create notable sediment compositional differences (most significantly feldspar and volcanic fragment content) that could be significant for long-term subsurface CO_2_ mineralization during CCS. Despite these differences, at a first order there appears to be globally-equitable distribution of high-quality potential storage resources on continental margins (Figs. 1 and 2; Ref ^11^).

Table A1 and Figure A2 show example functions we have used to describe stress and pressure profiles in two example basins – the Norwegian North Sea (NNS) and the Gulf of Mexico (GoM).


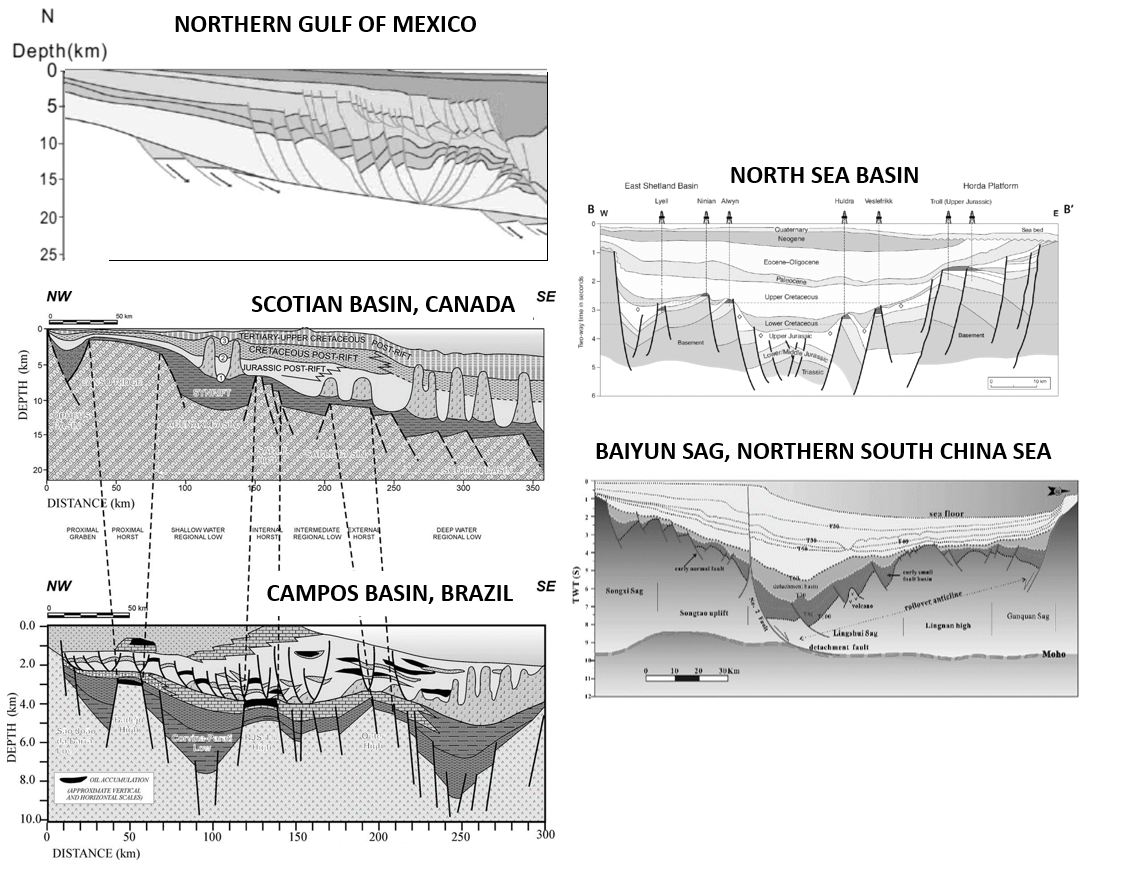


**Figure A1.** Comparison of previously interpreted schematic geologic cross sections from various passive extensional continental margins, showing broad stratigraphic and structural similarities: deep Mesozoic extensional faults, overlain by a progradational Late Mesozoic to Cenozoic section [Figure sources: Northern Gulf of Mexico^15^(https://creativecommons.org/licenses/by-sa/3.0/), North Sea^6^ (This figure is not covered by the CC BY license, © Geological Society of London, All rights reserved, used with permission), Scotian Basin^16^ (this figure is not covered by the CC BY license, © Geological Society of London, All rights reserved, used with permission), Campos Basin^16^ (this figure is not covered by the CC BY license, © Geological Society of London, All rights reserved, used with permission) and Baiyun Sag^17^].

| **Parameter** | **Norwegian North Sea case** | **Comments/Sources** | **Gulf of Mexico case** | **Comments/Sources** |
| --- | --- | --- | --- | --- |
| Seabed temperature | Assumed constant at 5^o^C | Ref^18^ | 15-25^o^C seasonally for inner-shelf water depths < 60 m | Ref^19^ |
| Geothermal gradient | 35^o^C/km | Ref^20^ | 23^o^C/km to 3,000 m increasing below to 34^o^C/km | Ref^21^ Figure 4.5; Ref^22^ |
| Brine density | 1020 kg/m^3^ to 1 km depth; increasing by 60 kg/km from 1 km downwards | Close to sea water density in shallow section; matches observations at Sleipner^20^ and Snøhvit | Average (N=66) of 1070 kg/m^3^ for Texas Miocene interval (1-3 km) | Supplementary Material^23^; produced waters database^24^ |
| Poisson’s ratio | 0.1 at surface increasing by 0.06/km with depth toward 0.4 at 5km | General match to well data used to estimate fracture pressure^6^ | 0.2 near surface increasing to 0.5 at 6 km depth | Ref^25^; Ref^26^ (Eqn. 7) |
| Bulk rock density | 2000 kg/m^3^ at surface; 2750 at 5km; constant gradient of 150 kg/km | General match to well data used to estimate vertical stress functions^6^ | 2000 kg/m^3^ near surface, reaching 2450 at 5 km depth. | Ref^25^ |

**Table A1.** Functions used to describe stress and pressure profiles in two example basins – NNS and GoM.

## A2 Offshore storage resources – methods and uncertainties

CO_2_ storage resources have been quantified in a number of national CO_2_ storage atlases^28,29,30,31^ and other studies^32,33,34^ which support the general conclusion that there are thousands of Gt of potential offshore storage space (static pore volumes) that are favorable for near-term CCS maturation in any given global offshore region. For example, the US NETL Atlas^31^ has offshore Saline Aquifer Formation (SAF) static storage capacity between 472 and 6433 billion metric tons, with a medium estimate of 2277 billion metric tons (Gt). These regional static volumetric assessments have necessarily used relatively simplified methods, and recent research indicates that capacity estimates that incorporate pressure limitations may be a factor of ten lower than estimates based on pore volume^35^. Furthermore, availability of potential resources for storage may also conflict with other users (including oilfield developments, wind-farm leases, and protected marine habitats), and areas with water depths greater than 1,000 meters (Ref^11^ Fig. 2) may slow development pace, leading to some practical restrictions. However, even with these limiting factors the continental margin basins have several hundreds of Gt of storage available for development in the 2020-2050 timeframe. Figure A3 shows an example of the storage resource for the Norwegian North Sea basin where 45.4 Gt of storage resources have been mapped^30^, with 16.8 Gt in the Cenozoic sequences and a further 28.6 Gt in the Mesozoic units. Equivalent capacity estimates for the Gulf of Mexico Cenozoic stratigraphy are around 558 Gt (Figure A4).

**Figure A2**. Basin depth functions for Norwegian North Sea with minimum stress data (S3) from Ref^6^ compared with GoM reservoir fluid pressures for Miocene age reservoirs in the Texas portion of the Gulf of Mexico^27^

Where national authorities have mapped the available resources for geological storage of CO_2_ in saline aquifers, these resource assessments generally estimate the available pore volume in porous geological rock units, scaled by a storage efficiency factor, ε, where ε is the fraction of the available pore space occupied by CO_2_. This storage efficiency factor attempts to capture the physical process of CO_2_ drainage into a water-wet brine-filled rock formation. Estimates for ε are typically around 4%, although local factors may render values between 1% and 6% with lower values generally corresponding to pressure-limited storage units. These estimates are founded on the principles of fluid dynamics whereby a buoyant non-wetting fluid displaces the in situ wetting fluid (brine) in a process controlled by the fluid mobility ratio and the gravity number^36,37^. We do not dispute the utility of using these storage efficiency factors to describe and quantify potential subsurface storage capacity but rather consider them to be insufficient for informing practical deployment of that global resource. Indeed, the long-running Sleipner project which demonstrates a storage efficiency of 5% after 22 years of injection provides some validation that these estimates for ε are operationally reasonable^38^.

Figure A5 shows an example of storage resource mapping from the Norwegian North Sea basin^30^, where we have added error bars based on the likely ranges for ε. When including these uncertainty ranges, there is still clearly a substantial resource available, with the total basin storage resource estimate lying between 10.5 Gt and 56 Gt (median = 45.4 Gt). The problem is not the availability of the storage resources, but rather the practical, temporal and economic means of exploiting the resource. A similar analysis for the GoM dataset (Figure A4) gives a range of 140 to 698 Gt around the median value of 558 Gt.


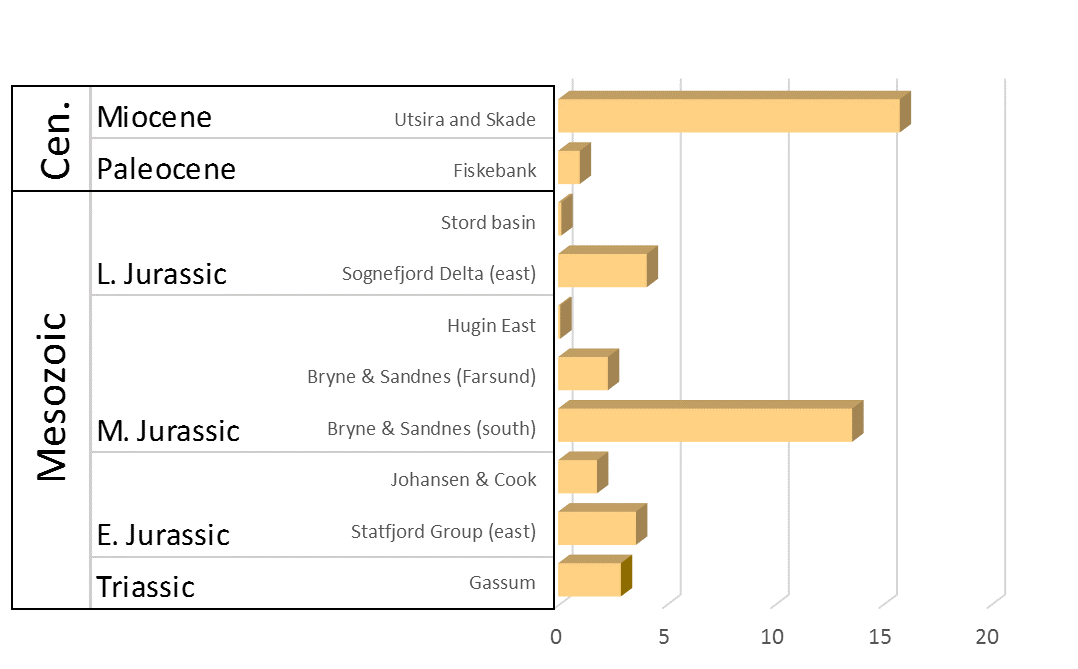


**Figure A3.** Stratigraphic disposition of CO_2_ storage resources for the Norwegian North Sea basin with capacity estimates from Ref^30^

**Figure A4.** Stratigraphic disposition of CO_2_ storage resources for the Cenozoic portion of Gulf of Mexico inner shelf basin with capacity estimates from Gulf Coast Carbon Center.

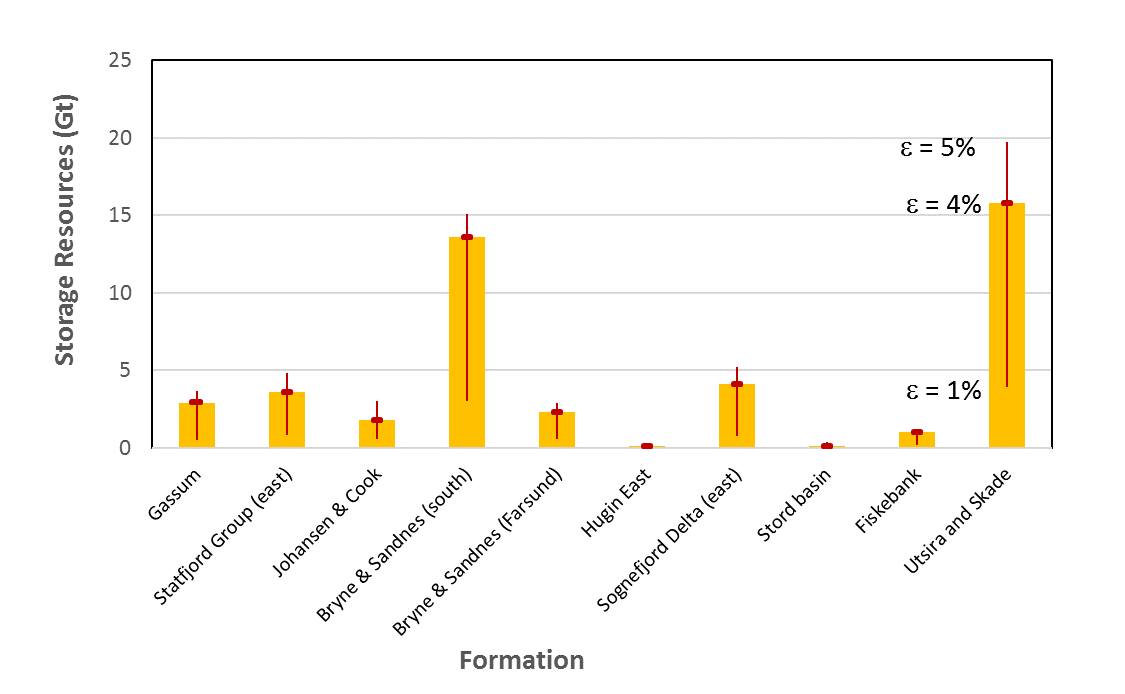


**Figure A5.** Example of mapped CO_2_ storage resources for the Norwegian North Sea basin^30^, with error bars based on the storage efficiency factor, ε. Actual values for ε are shown for the Utsira and Skade Formation case.

## A3 Derivation of functions for the basin fluid pressure analysis approach

We have addressed the problem of offshore storage resource assessment by using a generic approach based on pressure and stress trends in offshore sedimentary basins. For the case of non-infinite saline aquifers bounded by some set of structural or stratigraphic barriers, pressure will generally add a limiting factor to storage capacity estimates based on fluid dynamics of open systems. For any subsurface injection project, the geomechanical strength of the confined or semi-confined aquifer gives a practical limit to maximum allowable injection pressure, P_max_, which is defined with reference to the fracture pressure, P_frac_, of the relevant confining rock units. The definition of P_frac_ is complex and depends on the stress field, the borehole orientation and the *in-situ* rock properties^25,39,40^. The value for P_frac_ is generally close to (but not equal to) the minimum in situ stress component, σ_3_, of the stress tensor. For the purposes of this discussion we use an upper-bound estimate of the fracture pressure^40,41^, appropriate for vertical wells in sedimentary basins:

$P_{frac} =\frac{2\nu}{1-\nu}\left( \sigma_{V}-P \right)+P$ (1)

where ν is Poisons ratio, σ_V_ is the vertical (overburden) stress and P is the pore pressure.

For any specific CO_2_ storage project, we can then define the available pressure range for injection:

ΔP = P_max_ - P_init_ ≈ P_frac_ - P_init_ (2)

To define the initial pressure condition, we need to have some knowledge of the basin history. For this analysis we consider two well-known basins – the North Sea (Norway) and the Gulf Coast (USA) – where we have a good knowledge base from several decades of oil and gas exploration and production. Figure A2 (and Fig. 3 main paper^11^) shows the general situation in the Norwegian North Sea and Gulf of Mexico. Most saline aquifer formations are normally pressured (i.e. close to the hydrostatic pressure, P_hydro_) in the upper 2-3 km interval. In these intervals P_init_ ≈ P_hydro_. The deeper units which tend to be over-pressured, will then have P_init_ > P_hydro_. A further important factor for definition of P_init_ is that pressure depletion in oil and gas fields may cause depleted initial pressures in saline aquifer formations which are hydraulically connected to the hydrocarbon resource. These production-related pressure depletions may negate initial overpressures or even take initial pressures below P_hydro_. We will address the interactions between emerging developments of saline storage resources and hydrocarbon resource developments in the subsequent discussion.

The performance of a CO_2_ injection well can be summarized by the Injectivity Index, II_CO2_, which for the simplest case is given by:

${II}_{CO2}=\frac{q}{\left( p_{fbhp}- p_{res} \right)}$ (3)

where q is the well flow rate and p_fbhp_ is the flowing bottom-hole pressure and p_res_ is the far-field reservoir pressure. In practice, several other terms may need to be included in the function to account for near-wellbore effects and pressure and temperature gradients within the wellbore^42,43^. Injectivity may also vary as a function of time due to, for example, near-wellbore geochemical and geomechanical processes and long-term trends in far-field reservoir pressure^44^.

To generalize the long-term performance of an injection well in a saline aquifer we adapt the equation for radial flow around a wellbore^42^ to give an integrated function for the flow rate over the lifetime of the injection well in the time interval *i* to *f*:

$\int_{i}^{f} q_{t}=\frac{2\pi k_{a}h_{a}}{\mu\ln\left( \frac{r_{e}}{r_{w}} \right)}\left[ \int_{i}^{f} \left( p_{well}-p_{res} \right) \right]$ (4)

where k_a_ is the permeability of the aquifer formation, h_a_ is the height of the injection well interval, μ is the CO_2_ viscosity, r_e_ is the effective radius of the reservoir unit and r_w_ is the radius of the well. Generalizing this function and adding a flux term, F_b_, to represent a flux boundary condition for the injection unit, we have:

$V_{injected}=I_{C}\int_{i}^{f} \left( p_{well}-p_{res} \right)-F_{b}$ (5)

where V_injected_ is the total volume injected over the project lifetime and I_c_ is a constant equivalent to the mean Injectivity index. For a closed saline aquifer unit with no-flow boundary conditions (such as a sealed fault block), F_b_ = 0. If there is some pressure dissipation from the saline aquifer formation, F_b_ is positive, while a case with some brine influx into the storage unit has negative F_b_. It is assumed that F_b_ is normally a small factor compared to the injectivity term. However, for the case of an infinite aquifer with no pressure boundary limitation, F_b_ could be large or even dominant. The integral of reservoir pressure with time will be a function of the formation properties and the dimensions of the storage unit (main paper^11^ Fig. 4). This pressure function would normally be estimated using reservoir simulation of numerical models of the complex basin architecture (including effects faults and internal rock heterogeneity). However, general experience and knowledge of pressure propagation in porous media, suggests that the pressure will follow a characteristic function of time, based on pressure transient analysis^45,46^, where the dimensionless pressure function has the form:

$p_{D}\left( t_{D} \right)= \frac{1}{2}\ln\left( \frac{4t_{D}}{\gamma} \right)$ (6)

where p_D_ is dimensionless pressure, t_D_ is dimensionless time and γ is 1.781 (related to Euler’s constant). The coefficient of ½ may lie above or below this value depending on the reservoir boundary conditions (open, closed, or transient), but is assumed to be ½ for this analysis. Effects of compressibility of CO_2_ are omitted in this analysis, being a short-term transient effect, while the compressibility of the total fluid-rock system is embedded in the p_D_ function. To apply this equation in real dimensions for a pressure build-up case we can define:

$p_{res}\left( t \right)=p_{init}+A p_{D}$(t_D_) (7)

where A is a scaling parameter (related to the reservoir characteristics). Combining equations 5 and 7, we can obtain a general equation for the storage volume as a function the pressure bounds:

$V_{project}=I_{C} \left[ p_{well}-p_{init}+\int_{i}^{f} Ap_{D}\left( t_{D} \right) \right]+F_{b}$ (8)

It should be emphasized that Equation 8 assumes a constant injection pressure and constant injectivity – simplifying assumptions appropriate for screening prospective projects. With more complex operational variables, numerical reservoir simulation can be used to more accurately assess injection volumes as a function of variable pressure gradients. At the project screening stage, parameters for Equation 8 can be estimated using regional basin data and initial estimates of storage unit geometry and formation permeability. Volumes are converted to mass using estimates for the mean in situ density. For more detailed project designs, high-resolution digitized reservoir simulation models would be needed.

To illustrate the utility of the pressure-based method for estimation of CO_2_ storage volumes, we apply Equations 6-8 to the known pressure history at the Snøhvit CO_2_ injection project offshore Norway. Here we consider only a 3-year injection period for the Tubåen reservoir, which was followed by a second injection phase into a different shallower reservoir unit^47^. Here the bottom-hole pressure (BHP) is measured at a gauge 800m above the reservoir^47^ allowing BHP to be estimated accurately. We first re-scaled the dimensionless pressure function to the observed pressure history, assuming p_init_ = 290 bars and A = 34 (with time measured in months), to give the function P_res_ (Fig. A6) using Equation 7. Assuming a constant P_well_ of 380 bars, we then calculate the volume injected assuming I_c_ = 40 m^3^/day/bar (the expected injectivity prior to project start-up^48^), using Equation 8. The result is 1.13 Mt injected over the 34-month period (June 2008 to April 2011). This is slightly higher than the actual injected volume of 1.09 Mt, a reasonable error given that actual injection history was affected by stoppages. Also, we assume F_b_ is negligible for this example.


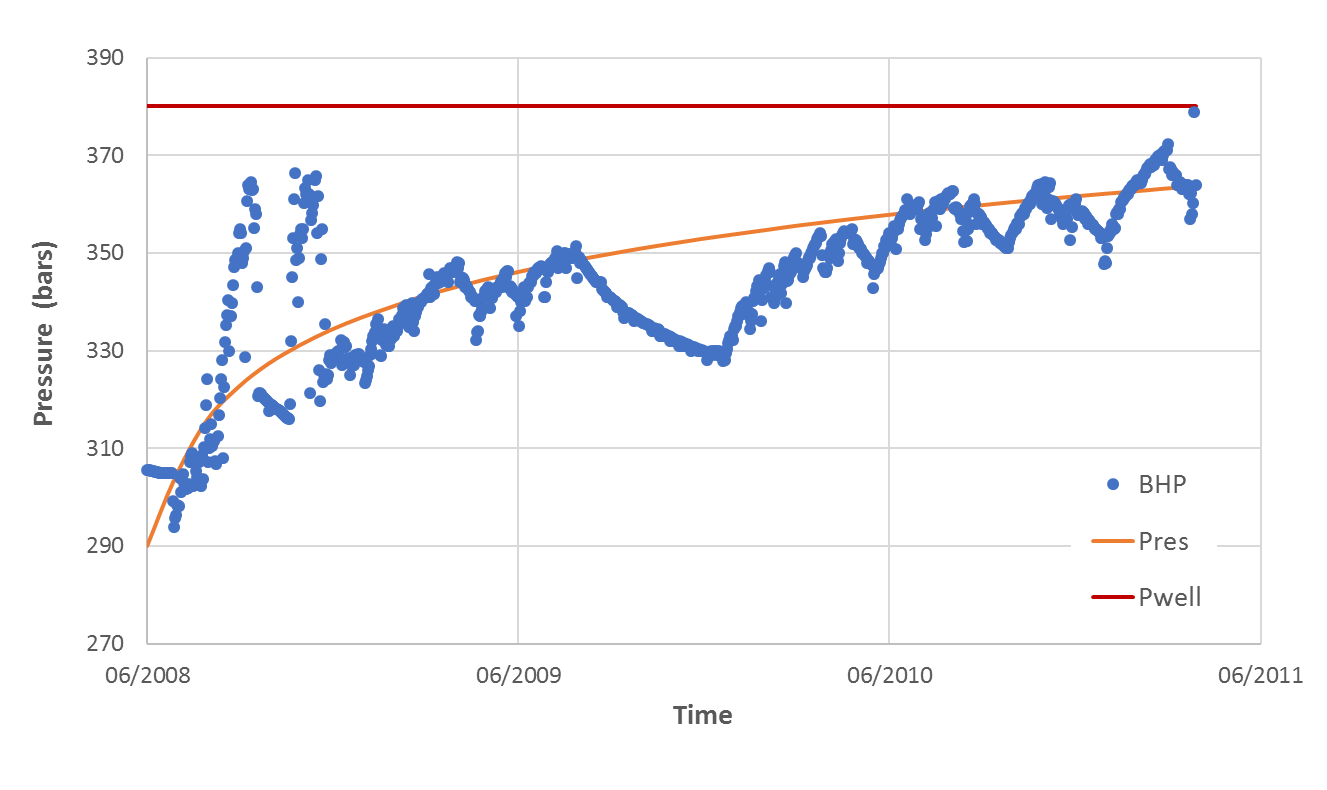


**Figure A6.** Pressure functions used to make a simplified storage volume estimate for the Snøhvit injection case.

## A4 Historic CO_2_ injection well data

Summary statistics for published industrial-scale SAF injector wells (used as basis for future injection-rate scenarios) are shown in Tables A2 and A3, with the statistical distributions of the data alongside the model scenarios used in the paper shown in Figure A7. The dataset comprises 60 years of injection data from 9 wells, with a mean rate of 0.532+0.271 Mt/year. For the offshore wells the mean rate is 0.695+0.222. We infer that 0.7Mt/year/well is a realistic average rate for the offshore case. The P90-P10 range is 0.330-1.059, which has been used to define confidence intervals the future well scenarios (P90 refers to 90% probability of exceedance). Higher injection rates are technically possible (of order 2 Mtpa per well) and future technology development is expected to lead to a future mean rate of closer to 1.0 Mt/year/well. Figure A8 shows the pressure functions for the four model scenarios (Main paper^11^) chosen to represent the range of expected behavior.

| **Project** | **Sample  (injection years)** | **Injection rate per well (Mt/year)** | **Equiv. rate  (t/hour)** | **Estimated formation permeability (Darcy) / porosity** |
| --- | --- | --- | --- | --- |
| Sleipner (peak) | 1 | 1.01 | 115 | 1-8 / 0.36 |
| Sleipner (mean) ^16, 34^ | 21 | 0.85 | 97 |  |
| Snøhvit-Stø (mean) | 8 | 0.61 | 70 | 0.01-0.8 / 0.12-0.20 |
| Snøhvit-Tub (mean) ^47,49^ | 3 | 0.33 | 38 |  |
| Quest (mean) ^50^ | 3 | 0.58 | 66 | 0.1 / 0.17 |
| Decatur (mean) ^51,52^ | 1 | 0.33 | 38 | 0.185 / 0.20 |
| In Salah (mean) ^53,54^ | 18 | 0.21 | 24 | 0.01 / 0.18 |

**Table A2.** Injection rates and formation summary data from industrial-scale SAF storage projects in operation.

|  | **All wells** | **Offshore only** |
| --- | --- | --- |
| N | 60 | 34 |
| Mean | 0,532 | 0,695 |
| Median | 0,583 | 0,725 |
| S.D. | 0,271 | 0,222 |
| 1.645 times S.D. | 0,446 | 0,364 |
| P90 rate | 0,086 | 0,330 |
| P10 rate | 0,978 | 1,059 |

**Table A3.** Statistics of injection rate data for all wells and for offshore projects only (P90 and P10 give the 90% confidence interval)

**Figure A7.** (A) Box and whisker plots of rate distributions data from all storage projects in operation and (B) Similar plot for offshore wells compared with mean rates for model scenarios (yellow symbols).


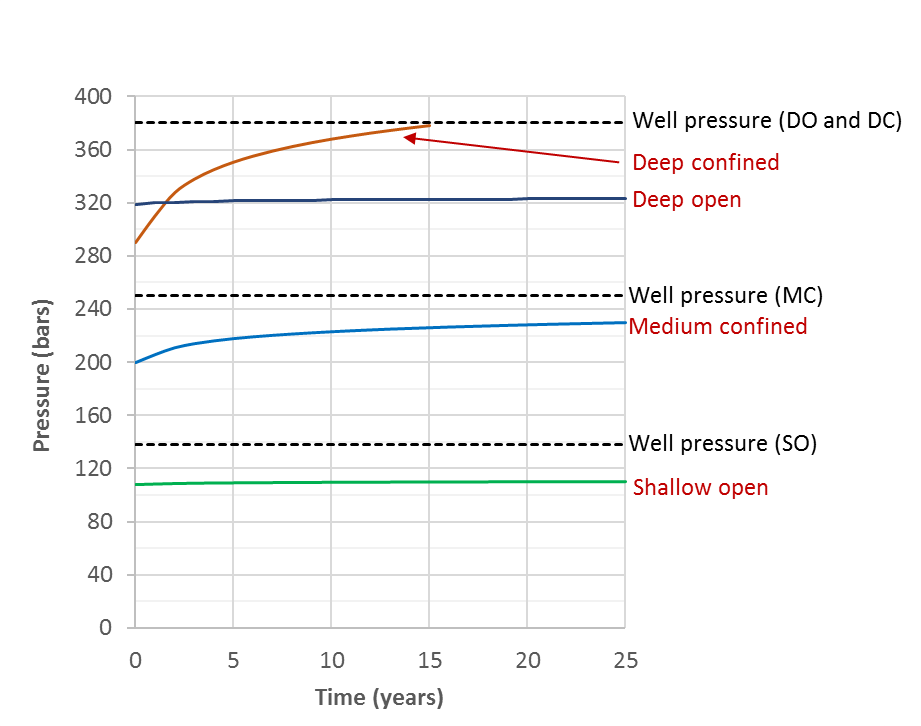


**Figure A8.** Pressure functions for modelled scenarios (coloured lines are reservoir pressure; dotted lines are well pressure, BHP).

## A5 Growth curve for GCS injection wells – methods and uncertainties

All new technologies experience an early steep growth curve, typically exponential growth for decades, until they become routine. GCS is at the beginning of that growth curve^55^. This early period is one of rapid innovation and cost reduction and development of economies of scale. This concept of technology market penetration and commercial materiality has been well studied for different technologies, with one recent analysis comparing anticipated CCS development with historic industrial deployment examples^56^. Given a current global CO_2_ storage rate of <10 Mt CO_2_ storage per year and an anticipated future rate of a few Gtpa by 2050, a scale up of >2 orders of magnitude over decades is required. This is not dissimilar from the historic performance of other technologies and can be considered as a reasonable initial expectation given a favorable economic environment, which admittedly has yet to fully mature (although successful tax and credit trading schemes are emerging and being implemented in some regions).

A useful perspective for evaluating realistic CCS deployment timelines is to look at historic hydrocarbon development. While some aspects of CCS deployment may differ from hydrocarbon extraction, sufficient experience with large-scale hydrocarbon resource development exists to anticipate the pace of CCS injection well deployment. In many ways, the similarities of the two activities and decades of prior experience suggest CCS could likely improve on early hydrocarbon industry rates of well deployment, although that is only speculation, so we take prior technical and temporal development experience as a minimum bound on CO_2_ injection well deployment rate. The subsequent analysis assumes economic viability (costs are not considered, but favorable economics are recognized as required to drive CCS activity), and no additional technological or engineering developments are considered (existing project technology considered sufficient, a very conservative assumption). The analysis simply uses historic well deployment data and injection rates anticipated based on current experience to confirm CCS viability on the needed decadal timeline already established.

These curves exhibit a characteristic shape related to their hydrocarbon extraction context, which relates to basin hydrocarbon ‘creaming’ curves^57,58,59^. The different maximum plateau values relate to overall basin size, total hydrocarbon resource retained, and degree of petroleum exploration. It is unknown if long-term CCS will emulate this overall temporal or spatial evolution of hydrocarbon development, but considerations of regional pressure presented in the main paper are consistent with creaming concepts, as the pressure resource of an area is likely to be exhausted before the total available rock pore volume is.

We have used historic well data to speculate on what the development of offshore CCS could look like in the future (Main paper^11^, Fig. 6). The more granular and relatively small-scale example of historic well deployment for hydrocarbon extraction in the inner shelf of the Texas portion of the Gulf of Mexico (<10.5 miles offshore) may be most appropriate for considering initial short-term CCS development in a region. Offshore well drilling activity began in this region in approximately 1950 and about 1,800 total wells were drilled in around 60 years. This can be considered representative of an aggressive industrial development setting on a continental margin, but not atypical in a global sense in terms of aerial extent, water depth, or geology, and is the most conservative well development model for CCS deployment (compared with the well data for the larger areas of the Norwegian North Sea and the US Gulf of Mexico).

Table A4 shows selected well-development scenarios based on the three historic datasets, used to illustrate the range in well rates and corresponding storage volumes achieved by 2050. Using the three regional and incrementally larger well-development models (Texas, Norway, GoM), a constant average well injection rate is applied to the well development timeline to calculate the incremental and cumulative CO_2_ stored. The ‘Texas model’ results indicate that global goals are not achieved in any scenario (varying injection rate and/or number of wells). Unrealistically high individual well rates (>4 Mtpa) would be needed to achieve total injection rates of 7 Gtpa in 2050. The Norwegian model results are more promising, in that five ‘Norway region’ models could achieve the goal of 7 Gtpa in 2050, assuming realistic average injection rates 0.67 Mtpa for 10,415 wells. The ‘Gulf of Mexico’ model results represent the most aggressive development case considered. Reasonable individual well injection rates allow the goal of 7 Gtpa in 2050 to be exceeded and could exceed 100 Gt cumulative storage. Of course, it is unlikely that one region will develop this aggressively to achieve needed reductions by itself.

| **2020+ Scenario** | **Offshore Well model** | **Number of regions** | **Avg. Well Inj. Rate (Mt/yr)** | **# active wells in 2050** | **Incremental Rate in 2050 (Mt/yr)** | **Cumulative Mass in 2050 (Mt CO_2_)** | **Comment** |
| --- | --- | --- | --- | --- | --- | --- | --- |
| A | Texas | 1 | 0.7* | 345 | 242 | 1,781 | Goals not met |
| B | Texas | 5 | 0.7* | 1,725 | 1,208 | 8,904 | Goals not met |
| C | Texas | 5 | 4.058 | 1,725 | 7,000* | 51,617 | Incremental rate goals met, but not cumulative; injection rate high |
| D | Norway | 1 | 0.7 | 2,083 | 1,458 | 15,243 | Goals not met |
| E | Norway | 1 | 3.36 | 2,083 | 7,000* | 73,164 | Incremental rate goals met, but not cumulative; injection rate very high |
| **F** | Norway | **5** | **0.672** | **10,415** | **7,000*** | **73,164** | **Most plausible** |
| G | GoM | 1 | 0.7* | 17,155 | 12,009 | 116,523 | Unlikely one region will develop this aggressively; Incremental goal exceeded; Close to cumulative goal |
| H | GoM | 1 | 0.408 | 17,155 | 7,000* | 67,916 | Injection rate low, not cost effective; Cumulative goal not met |

**Table A4:** Eight possible scenarios for CCS development on continental shelves based on historic examples of hydrocarbon well development from three different offshore regions: Texas, Norway, and Gulf of Mexico (GoM). Each scenario uses one of 3 historically-based models for well development (Main paper Fig. 6) and considers constant average individual well injection rates and three metrics for the year 2050: the total number of active wells (based on historical development, modified for 25-year lifespan), the total rate of CO_2_ injection at that time, and the cumulative mass of CO_2_ injected. Values with an asterisk are prescribed in each scenario, with the others being dependent values. Column 6 is the product of values in columns 4 and 5. Scenario F seems most plausible for giga-ton scale deployment, requiring a well development model similar to historic Norwegian hydrocarbon exploitation to be applied for CCS in 5-7 regions globally, with a reasonable mean well injection rate of approximately 0.67 Mt/yr.

For an additional perspective on a fully mature global injection scenario, from 1968 to 2001 (33 years), there were globally on average around 1,500 new hydrocarbon exploration wells drilled per year outside of the US and Canada in all water depths, with a peak of around 2,200 and a low of 800 per year^60^. Globally, 17,700 wells were drilled in shallow water since 1940 (similar to Scenario G, Table A4). This represents a mature global hydrocarbon development stage, so is arguably similar to what mature GCS could achieve eventually. Not all of those wells were successful (encountered economic hydrocarbons), so a failure rate may need to be considered. However, given the current maturity of basin knowledge, new CO_2_ storage injectors should have a high success rate. Assuming (for a CCS scenario) that each of the projected 1,500 wells per year achieves an average injection rate of 0.5 Mt/yr (allowing for many unsuccessful wells), this equates to increasing global CO_2_ storage by 750 Mt/yr each year. The cumulative storage of this mature activity over a decade (from say 2050 to 2060) would be to store over 40 Gt of CO_2_. Clearly this would require development of a global CCS industry comparable to the size of the hydrocarbon exploration industry in about half the time. Challenging, but credible. Such industrial growth could drive investment, employment, and long-term prosperity in many regions, but would need to be accompanied by favorable economic markets/incentives such as globally validated and tradable CO_2_ credits.

## References (Appendix)

1. Haq, B.U., Hardenbol, J., & Vail, P.R. Chronology of Fluctuating Sea Levels Since the Triassic, Science: Vol. 235 (4793), 1156-1167 DOI: 10.1126/science.235.4793.1156 (1987).
2. Vail, P.R. Seismic Stratigraphy Interpretation Using Sequence Stratigraphy: Part 1: Seismic Stratigraphy Interpretation Procedure, In: A. W. Bally (Ed.) *AAPG Studies in Geology #27*, volume 1: Atlas of Seismic Stratigraphy (1987).
3. Van Wagoner, J.C., H.W. Posamentier, R.M. Mitchum, P.R. Vail, J.F. Sarg, T.S. Loutit, and J. Hardenbol. An overview of the fundamentals of sequence stratigraphy and key definitions, SEP Special Publication No. 42, The Society of Economic Paleontologists and Mineralogists (1988).
4. Weeks L.G. Petroleum Resources Potential of Continental Margins, In: Burk C.A., Drake C.L. (eds) *The Geology of Continental Margins.* Springer, Berlin, Heidelberg (1974).
5. Magoon, L.B., and W.G. Dow, The petroleum system, in, Magoon, L.B., and W.G. Dow, eds, The Petroleum System-from Source to Trap: *AAPG Memoir 60*, p. 3-24 (1994)
6. Bolås, H. M. N., & Hermanrud, C. Hydrocarbon leakage processes and trap retention capacities offshore Norway. *Petroleum Geoscience,* 9(4), 321-332 (2003).
7. Osborne, M. J., & Swarbrick, R. E. Mechanisms for generating overpressure in sedimentary basins: A reevaluation. *AAPG bulletin,* 81(6), 1023-1041 (1997).
8. Schowalter, T.T. Mechanics of secondary hydrocarbon migration and entrapment, *AAPG Bulletin,* 63(5): 723-760 (1979).
9. Demaison, G. & Huizinga, B.J. Genetic classification of petroleum systems, *AAPG Bulletin,* 75(10): 1626-1643 (1991).
10. Zoback, M.L. First‐ and second‐order patterns of stress in the lithosphere: The World Stress Map Project, *JGR Solid Earth,* V97(B8): 11703-11728, <https://doi.org/10.1029/92JB00132> (1992)
11. Ringrose, P.S. & Meckel, T.A. Maturing global CO2 storage resources on offshore continental margins to achieve 2DS emissions reductions (this submission, 2019).
12. Grollimund, B. & Zoback, M.D. Impact of glacially-induced stress changes on fault seal integrity offshore Norway, *AAPG Bulletin*, 87(3): 493-506 (2003).
13. Plaza-Faverola, A., Buenz, S. Mienert, J. Repeated fluid expulsion through sub-seabed chimneys offshore Norway in response to glacial cycles, *Earth and Planetary Science Letters,* 305: 297-308 (2011).
14. Longley, I.M., C. Buessenschuett, L. Clydsdale, C.J. Cubitt, C.J., Davis, R.C., Johnson, M.K., Marshall, N.M., Murray, A.P., Somerville, R., Spry, T.B and N.B. Thompson, 2003, The North West Shelf of Australia – A Woodside Perspective, AAPG Search and Discovery article #10041.
15. Xie, F., Q. Wu, L. Wang, Z. Shi, C. Zhang, B. Liu, C. Wang, Z. Shu, and H. Di, 2017, Passive continental margin basins and the controls on the formation of evaporates: a case study of the Gulf of Mexico Basin, Carbonates Evaporites, DOI 10.1007/s13146-017-0404-z.
16. Mohriak, W.U., and S. Leroy, 2013, Architecture of rifted continental margins and break-up evolution: insights from the South Atlantic, North Atlantic and Red Sea-Gulf of Aden conjugate margins, In: Mohriak, W. U., Danforth, A., Post, P. J., Brown, D. E., Tari, G. C., Nemcˇok, M.&Sinha, S. T. (eds) 2013. Conjugate Divergent Margins. Geological Society, London, Special Publications, 369, 497–535.
17. Tang, L., J. Ren, K. Mcintosh, X. Pang, C. Lei, and Y. Zhao, 2018, The structure and evolution of deepwater basins in the distal margin of the northern South China Sea and their implications for the formation of the continental margin, Marine and Petroleum Geology, 92: 234-254, https://doi.org/10.1016/j.marpetgeo.2018.02.032
18. Nooner, S. L., Eiken, O., Hermanrud, C., Sasagawa, G. S., Stenvold, T., & Zumberge, M. A. Constraints on the *in situ* density of CO2 within the Utsira formation from time-lapse seafloor gravity measurements. *International Journal of Greenhouse Gas Control,* 1(2), 198-214 (2007).
19. Turner R.E., Rabalais N.N. & Justić D. Trends in summer bottom-water temperatures on the northern Gulf of Mexico continental shelf from 1985 to 2015. *PLoS ONE* 12(9): e0184350. https://doi.org/10.1371/journal.pone.0184350 (2017).
20. Singh, V. P., Cavanagh, A., Hansen, H., Nazarian, B., Iding, M., & Ringrose, P. S. Reservoir modeling of CO2 plume behavior calibrated against monitoring data from Sleipner, Norway. In SPE annual technical conference and exhibition. *Society of Petroleum Engineers (*2010).
21. Meckel, T.A., Nicholson A.J. & Treviño, R.H. Capillary aspects of fault-seal capacity for CO_2_ storage, Lower Miocene, Texas Gulf of Mexico, In: Treviño, R.H., and T.A. Meckel, Eds., Geological CO_2_ sequestration atlas of Miocene strata, offshore Texas state waters, Bureau of Economic Geology, Report of Investigations No. 283 (The University of Texas at Austin, 2018).
22. Morris, S., B. Vestal, K. O’Neill, M. Moretti, C. Franco, N. Hitchings, J. Zhang, and J.D. Grace, The pore pressure regime of the northern Gulf of Mexico: Geostatistical estimation and regional controls, *AAPG Bulletin,* 99(1): 91-118 (2015).
23. Yang, C., R.H. Trevino, T. Zhang, K.D. Romanak, K. Wallace, J. Lu, P.J. Mickler, & S.D. Hovorka, Regional assessment of CO_2_-solubility trapping potential: A case study of the coastal and offshore Texas Miocene interval, *Environmental Science and Technology,* 48: 8275-8282, dx.doi.org/10.1021/es502152y (2014).
24. U.S. Geological Survey World Petroleum Assessment 2000, Digital Data Series 60, <https://energy.usgs.gov/OilGas/AssessmentsData/WorldPetroleumAssessment.aspx> (2000)
25. Eaton, B.A. Fracture gradient prediction and its application in oilfield operations, Society of Petroleum Engineers, SPE-2163-PA, <https://doi.org/10.2118/2163-PA> (1969).
26. Marbun, B.T.H., A.N. Corina, G.V. Arimbawa, R. Aristya, S. Purwito, and A.F. Hardama, A new approaching method to estimate fracture gradient by correcting Matthew-Kelley and Eaton’s stress ratio, *Journal of Petroleum Science and Engineering,* 135: 261-267 (2015).
27. Treviño, R.H., & T.A. Meckel (Eds.) Geological CO_2_ sequestration atlas of Miocene strata, offshore Texas state waters, Bureau of Economic Geology, Report of Investigations No. 283, The University of Texas at Austin, 74 p. (2017).
28. Vangkilde-Pedersen, T., Anthonsen, K. L., Smith, N., Kirk, K., Neele, F., van der Meer, B., ... & Hendriks, C. Assessing European capacity for geological storage of carbon dioxide–the EU GeoCapacity project*. Energy Procedia,* 1(1), 2663-2670 (2009).
29. Bentham, M., Mallows, T., Lowndes, J., & Green, A. CO2 Storage Evaluation Database (CO2 Stored): the UK's online storage atlas. *Energy Procedia,* 63, 5103-5113 (2014).
30. Halland, E. K., Mujezinovic, J., & Riis, F. CO_2_ Storage Atlas: Norwegian Continental Shelf, Norwegian Petroleum Directorate, PO Box 600, NO-4003 Stavanger, Norway, 2014. URL http://www.npd. no/en/Publications/Reports/Compiled-CO2-atlas (2014).
31. NETL, 2015. Carbon Storage Atlas, 5^th^ Edn. <https://www.netl.doe.gov/>
32. Hendriks, C., and W. Graus, Global Carbon Dioxide Storage Potential and Costs, In: EEP-02001 Ecofys (2004).
33. Metz, B., Davidson, O., De Coninck, H., Loos, M. and Meyer, L. *Carbon dioxide capture and storage:* Working Group III of the Intergovernmental Panel on Climate Change. Cambridge, United Kingdom and New York, NY, USA 442 (2005).
34. Dooley, J.J., Estimating the supply and demand for deep geologic CO_2_ storage capacity over the course of the 21^st^ century: A meta-analysis of the literature, *Energy Procedia*, *37*, 5141-5150 (2013).
35. Gorecki, C. D., Sorensen, J. A., Bremer, J. M., Knudsen, D., Smith, S. A., Steadman, E. N., & Harju, J. A. Development of storage coefficients for determining the effective CO_2_ storage resource in deep saline formations. In SPE International Conference on CO2 Capture, Storage, and Utilization. Society of Petroleum Engineers (2009).
36. Nordbotten, J. M., & Celia, M. A. Similarity solutions for fluid injection into confined aquifers. *Journal of Fluid Mechanics,* 561, 307-327 (2006).
37. Okwen, R. T., Stewart, M. T., & Cunningham, J. A. Analytical solution for estimating storage efficiency of geologic sequestration of CO2. *International Journal of Greenhouse Gas Control,* 4(1), 102-107 (2010).
38. Ringrose, P. S., The CCS hub in Norway: some insights from 22 years of saline aquifer storage. *Energy Procedia,* 146, 166-172 (2018).
39. Zoback, M.D. Reservoir Geomechanics. Cambridge University Press, Cambridge, UK (2007).
40. Bohloli, B., Ringrose, P., Grande, L., & Nazarian, B. Determination of the fracture pressure from CO2 injection time-series datasets. *International Journal of Greenhouse Gas Control,* 61, 85-93 (2017).
41. Zhang, J. Pore pressure prediction from well logs: Methods, modifications, and new approaches. *Earth-Science Reviews,* 108(1-2), 50-63 (2011).
42. Golan, M., & Whitson, C. H. Well performance (p. 469). Englewood Cliffs, New Jersey: Prentice Hall (1991).
43. Ringrose, P., Greenberg, S., Whittaker, S., Nazarian, B., & Oye, V. Building confidence in CO2 storage using reference datasets from demonstration projects. *Energy Procedia,* 114, 3547-3557 (2017).
44. Pawar, R. J., Bromhal, G. S., Carey, J. W., Foxall, W., Korre, A., Ringrose, P. S., ... & White, J. A. Recent advances in risk assessment and risk management of geologic CO 2 storage. *International Journal of Greenhouse Gas Control,* 40, 292-311 (2015).
45. Miller, C. C., Dyes, A. B., & Hutchinson Jr, C. A. The estimation of permeability and reservoir pressure from bottom hole pressure build-up characteristics. *Journal of Petroleum Technology*, *2*(04), 91-104 (1950).
46. Dake, L. P. The practice of reservoir engineering. *Developments in petroleum science*, 36, 311-458 (1994).
47. Hansen, O., Gilding, D., Nazarian, B., Osdal, B., Ringrose, P., Kristoffersen, J. B., ... & Hansen, H. Snøhvit: The history of injecting and storing 1 Mt CO2 in the fluvial Tubåen Fm. *Energy Procedia,* 37, 3565-3573 (2013).
48. Maldal, T., & Tappel, I. M. CO_2_ underground storage for Snøhvit gas field development. *Energy,* 29(9-10), 1403-1411 (2004).
49. Eiken, O., Ringrose, P., Hermanrud, C., Nazarian, B., Torp, T. A., & Høier, L. Lessons learned from 14 years of CCS operations: Sleipner, In Salah and Snøhvit. *Energy Procedia,* 4, 5541-5548 (2011).
50. Rock, L., O’Brien, S., Tessarolo, S., Duer, J., Bacci, V. O., Hirst, B., ... & Halladay, A. The Quest CCS Project: 1st Year Review Post Start of Injection. *Energy Procedia*, *114*, 5320-5328 (2017).
51. Finley, R. J., Frailey, S. M., Leetaru, H. E., Senel, O., Couëslan, M. L., & Scott, M. Early operational experience at a one-million tonne CCS demonstration project, Decatur, Illinois, USA. *Energy Procedia*, *37*, 6149-6155 (2013).
52. Gollakota, S., & McDonald, S. Commercial-scale CCS project in Decatur, Illinois–construction status and operational plans for demonstration. *Energy Procedia*, *63*, 5986-5993 (2014).
53. Wright, I. W., Ringrose, P. S., Mathieson, A. S., & Eiken, O. An overview of active large-scale CO2 storage projects. In SPE International Conference on CO2 Capture, Storage, and Utilization. Society of Petroleum Engineers (2009).
54. Ringrose, P. S., Mathieson, A. S., Wright, I. W., Selama, F., Hansen, O., Bissell, R., ... & Midgley, J. The In Salah CO2 storage project: lessons learned and knowledge transfer. *Energy Procedia,* 37, 6226-6236 (2013).
55. Reiner, D.M., Learning through a portfolio of carbon capture and storage demonstration projects, *Nature Energy* Vol. 1, Article number: 15011 (2016).
56. [Kramer](https://www.nature.com/articles/462568a#auth-1), G.J. & Haigh, M. No quick switch to low-carbon energy, *Nature* 462: 568–569 (2009) [their Fig. 1]
57. Arps, J.J. & Roberts, T.G. Economics of drilling for Cretaceous oil on the east flank of the Denver-Julesburg Basin, *American Association of Petroleum Geologists Bulletin,* v. 42, no. 11, p. 2549-2566 (1958).
58. Meisner, J. & Demirmen, F. The creaming method: a bayesian procedure to forecast future oil and gas discoveries in mature exploration provinces: *Journal of the Royal Statistical Society*, v. 144, part A, p. 1-31 (1981).
59. Drew, LJ., and Lore, G.L, Field growth in the Gulf of Mexico--A progress report, in USGS Research on Energy Resources, 1992: *U.S. Geological Survey Circular 1074,* p. 22-23 (1992).
60. Attanasi, E.D., Freeman, P.A. & Glovier, J.A. Statistics of Petroleum Exploration in the World Outside the United States and Canada Through 2001, *USGS Circular 1288 (*2007).
